# Study on reservoir optimal operation based on coupled adaptive ε constraint and multi strategy improved Pelican algorithm

**DOI:** 10.1038/s41598-023-41447-0

**Published:** 2023-08-28

**Authors:** Ji He, Xiaoqi Guo, Songlin Wang, Haitao Chen, Fu-Xin Chai

**Affiliations:** 1 School of Water Resources, North China University of Water Resources and Electric Power, Zhengzhou, 450011 China; 2 China Institute of Water Resources and Hydropower Research, Research Center on Flood and Drought Disaster Reduction, Beijing, China

**Keywords:** Environmental sciences, Hydrology, Limnology

## Abstract

The optimal operation of reservoir groups is a strongly constrained, multi-stage, and high-dimensional optimization problem. In response to this issue, this article couples the standard Pelican optimization algorithm with adaptive ε constraint methods, and further improves the optimization performance of the algorithm by initializing the population with a good point set, reverse differential evolution, and optimal individual t-distribution perturbation strategy. Based on this, an improved Pelican algorithm coupled with adaptive ε constraint method is proposed (ε-IPOA). The performance of the algorithm was tested through 24 constraint testing functions to find the optimal ability and solve constraint optimization problems. The results showed that the algorithm has strong optimization ability and stable performance. In this paper, we select Sanmenxia and Xiaolangdi reservoirs as the research objects, establish the maximum peak-cutting model of terrace reservoirs, apply the ε-IPOA algorithm to solve the model, and compare it with the ε-POA (Pelican algorithm coupled with adaptive ε constraint method) and ε-DE (Differential Evolution Algorithm) algorithms, the results indicate that ε. The peak flow rate of the Huayuankou control point solved by the IPOA algorithm is 12,319 m^3^/s, which is much lower than the safe overflow flow rate of 22,000 m^3^/s at the Huayuankou control point, with a peak shaving rate of 44%, and other algorithms do not find effective solutions meeting the constraint conditions. This paper provides a new idea for solving the problem of flood control optimal operation of cascade reservoirs.

## Introduction

Flood is one of the most frequent and serious natural disasters faced by human beings. In China, floods occur frequently and extensively, seriously endangering people's lives and property^1^. In addition, 60–80% of the rainfall occurs mainly during the flood season, which exacerbates the severity of floods^2^. For example, a once-in-a-millennium extraordinarily heavy rainstorm hit Zhengzhou on July 20, 2021, causing millions of people to be affected and causing huge economic losses.

As an engineering measure, reservoirs play the role of storing flood water, reducing flood peaks and protecting downstream safety during the flood season^3^. Reservoir flood control operation, as a non-engineering measure corresponding to reservoirs, has always been a hot spot for research. There are often multiple reservoirs in the flood control system, and it is difficult for a single reservoir to fully play its role in flood control. However, there are complex hydrological and hydraulic connections and some strong constraints between reservoirs, making it more difficult to solve the reservoir flood control operation problem^4^.

In the past, scholars often used dynamic programming^5–7^ and linear programming^8^ to solve this problem, but as the number of reservoirs and the operation period in-crease, the convergence speed is slow and the problem of "dimensional disaster" occurs. The emergence of heuristic algorithms has solved this problem well, and some scholars have started to use heuristic algorithms to solve this problem. Cheng applied the chaotic genetic algorithm^9^ to the reservoir scheduling of hydropower station, and the convergence speed is much better than dynamic programming and standard genetic algorithm. He proposed an improved chaotic particle swarm algorithm^10^ based on logistic mapping to solve the reservoir flood control scheduling model. Chen coupled the Yin-Yang Firefly Algorithm algorithm^11^ and ε-constraint method to establish the three-reservoirs flood control scheduling model. Liu^12^ uses particle swarm optimization algorithm with coupled penalty function to solve the maximum peak clipping model of cascade reservoirs. Zhang^13^ proposed a genetic algorithm coupled with penalty function to solve the flood control operation problem of cascade reservoirs. Although the penalty function method is often used to solve constraint problems, the selection of penalty parameters is difficult to grasp and too small to play a penalty role; If it is too large, it will cause errors due to the influence of errors. Although intelligent algorithms have been widely used, they are too stochastic and easily fall into local optimal solutions, and the solution results are not stable.

In addition, simple optimization algorithms are difficult to solve the multi constraint and strong constraint problems of reservoir group flood control operation models, so constraint processing techniques need to be combined in the solving process to solve the relevant constraint conditions. ε Constraint method is a commonly used method for handling constraint conditions, however, research has shown that based on ε. The constrained optimization algorithm has achieved good results in handling constrained optimization problems, but further improvement is still needed in terms of convergence accuracy and robustness. Literature^14^ proposes an adaptive approach ε Constraint method, by improving constraint processing technology and adaptive settings ε, avoid falling into local optima, improve the robustness and search efficiency of the algorithm. The Pelican Optimization Algorithm (POA) is a novel heuristic algorithm pro-posed by Pavel Trojovský and Mohammad Dehghani^15^ in 2022, which simulates the behavior of pelicans hunting in nature. Experimental simulations comparing the POA algorithm with eight common algorithms such as Genetic Algorithm (GA), Differential Evolution (DE), and Grey Wolf Optimization Algorithm (GWO) in the literature demonstrate that the POA algorithm has high local search ability and convergence toward the global optimum. However, like other heuristic algorithms, it also has the drawback of too much randomness, which affects its exploration toward the global optimum. Therefore, this paper proposes a pelican algorithm (IPOA) based on good point set and reverse differential evolution. First, the inclusion of the good point set makes the initial population distribution more uniform and improves the diversity of the population^16^; second, the reverse differential evolution algorithm is incorporated to improve the diversity, convergence speed and optimality-seeking accuracy of the algorithm; finally, an adaptive t-distribution variation strategy is introduced for the optimal pelican individuals to avoid the algorithm from falling into local optimal solutions. The contributions of this article are as follows: to address the above issues, this paper proposes an IPOA algorithm with coupled adaptive ε-constraint method and the cec2006 test function set were used for simulation experiments. The experiment shows that the algorithm proposed in this paper not only finds the global optimal solution when solving constrained optimization problems, but also has good robustness compared to the original algorithm. Take ε-IPOA algorithm was applied to solve the flood control operation model of the Sanmenxia-Xiaolangdi cascade reservoir, and compared with other algorithms. The results indicate that, the application of ε-IPOA algorithm in reservoir operation problems has strong practicality, and the proposed scheme has a high peak clipping rate, which is more beneficial for downstream disaster prevention and reduction. This algorithm provides a new approach for solving the joint flood control operation of reservoir groups.

This paper is structured as follows: “Flood control operation model of cascade reservoirs” section shows the joint flood control and operation model for reservoir groups; “IPOA algorithm based on adaptive ε-constraint” section introduces the ε-IPOA algorithm and performs functions tests to prove its superiority; “Case analysis” section case study illustrates the results of joint operation of reservoir groups in the study area and discusses them; “Conclusion” section presents the conclusions of this paper.

## Flood control operation model of cascade reservoirs

### Objective function

In order to reduce the flood control burden of the downstream reservoirs and the safety of the downstream flood control points, the flood control operation model of cascade reservoirs is established based on the maximum peak clipping criterion. The maximum peak clipping criterion not only ensures that the maximum peak flow at the downstream control point is reduced, but also makes the discharge flow of the reservoir more uniform, reducing the flood risk of the basin.

**Figure 1:**
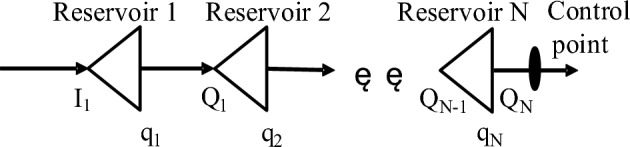
N-class reservoir generalization map.

As shown in Fig. 1, suppose that the basin flood control system has N cascade reservoirs, 1 flood control point, the inflow of the first reservoir and the interval flood flow between reservoirs are known, the number of dispatching periods is T, and the maximum peak clipping objective function of the downstream control point is as follows:

1 $$ob = \sum\limits_{t = 1}^{T} {\sum\limits_{n = 1}^{N} {\left( {q_{n,t} + Q_{n,t} } \right)} }^{2} \Delta t$$

where $q_{n,t}$ is the discharge flow of the n-th reservoir in the t period; $Q_{n,t}$ is the interval flood flow between the n-th reservoir and the (n + 1)-th reservoir; $\Delta t$ is the operation interval.

### Constraint condition

1. Water balance constraint
  2 $$V_{n,t} = V_{n,t - 1} + \left( {I_{n,t} - q_{n,t} } \right)\Delta t$$
2. Water level constraint
  3 $$Z_{n,\min } \le Z_{n,T} \le Z_{n,\max }$$
3. Initial water level, end of period water level constraint
  4 $$Z_{n,0} = Z_{n,begin}$$
  5 $$Z_{n,T} = Z_{n,end}$$
4. Discharge capacity constraint
  6 $$q_{n,t} \le q\left( {Z_{n,t} } \right)$$
5. Maximum safe discharge constraint
  7 $$q_{n,t} \le Q_{\max }$$
6. Non-negative constraint
  All the above variables are non-negative.
  Where $V_{n,t - 1}$, $V_{n,t}$ is the initial storage capacity and final storage capacity of the nth reservoir in the t period;$I_{n,t}$ is the inflow of the n-th reservoir in the t period;$q_{n,t}$ is the outflow of the n-th reservoir in the t period;$Q_{\max }$ is the maximum downstream flow allowed to ensure the safety of the downstream river;$q\left( {Z_{n,t} } \right)$ is the maximum discharge capacity of the nth reservoir when the initial water level is $Z_{n,t}$;$Z_{n,0}$ is the initial water level at the initial time of the n-th reservoir operation;$Z_{n,begin}$ is the starting water level of the n-th reservoir;$Z_{n,T}$ is the water level at the end of the n-th reservoir operation period.$Z_{n,end}$ is the expected water level at the end of the n-th reservoir operation period.

## IPOA algorithm based on adaptive ε-constraint

### POA algorithm

In the Pelican optimization algorithm, the behavior and strategies of the pelicans during attack and hunting were simulated to update candidate solutions. The hunting process is divided into two stages: approaching the prey (exploration stage) and flying on the water surface (development stage).

#### Initialization

8 $$X_{ij} = lb_{j} + rand*\left( {ubj - lbj} \right);i = 1,2,...,N;j = 1,2,...,D$$

In the equation, X_ij_ is the position of the j-th dimension of the i-th pelican; N is the number of populations; D is the dimension of the decision variable; lb and ub are the lower and upper bounds of the decision variables respectively.

#### Phase 1: Exploration phase

In the first stage, the pelican determines the location of its prey and then moves towards this designated area. The mathematical model is as follows:

9 $$X_{i,j}^{new1} = \left\{ {\begin{array}{ll} {X_{i,j} + rand*\left( {P_{j} - I*X_{i,j} } \right)}, & \quad F_{p} < F_{i} \\ {X_{i,j} + rand*\left( {X_{i,j} - P_{j} } \right)}, & \quad else \\ \end{array} } \right.$$

In the formula, $X_{i,j}^{new1}$ is the position of the j-th dimension of the i-th pelican based on the first stage update; I is a random integer of 1 or 2; P_j_ is the position of the prey in the jth dimension; F_p_ is the objective function value of the prey; F_i_ is the objective function value of the i-th pelican.

#### Phase 2: Development phase

10 $$X_{i,j}^{new2} = X_{i,j} + R*\left(1 - \frac{iter}{{Maxiter}}\right)*\left( {2*rand - 1} \right)*X_{i,j}$$

In the formula, $X_{i,j}^{new2}$ is the position of the j-th dimension of the i-th pelican based on the second stage update; R is a random integer of 0 or 2; iter is the number of current iterations; Maxiter is the maximum number of iterations.

### IPOA algorithm

In order to improve the performance of the POA algorithm, the following improvements are made in this paper on the basis of the POA algorithm.

#### Goodpoint set principle

The standard POA algorithm uses a random method to initialize the pelican population, which is highly randomized and some better pelican individuals are easily ignored. In this paper, we adopt the good point set theory proposed by Chinese mathematician Hua Luogeng to initialize the pelican population. Good point set is an effective method that can select points uniformly, and compared with the random method, the points taken using the good point set method can be more uniformly distributed in the search space. The literature^17,18^ demonstrated that the population initialized with good point set theory is more uniform and can increase the diversity of the population during the initialization process. The formula is as follows:

11 $$\begin{aligned} x_{ij} & = i*2\cos \left( {\frac{2\pi j}{p}} \right) \hfill \\ x{\prime}_{ij} & = lb_{j} + \bmod (x_{ij} ,1)*\left( {ub_{j} - lb_{j} } \right) \hfill \\ \end{aligned}$$

where p is the smallest prime number satisfying $\frac{p - 3}{2} \ge D$;

#### Fusion of reverse differential evolution strategy

The main idea of the reverse learning strategy^19^ is that when searching for the optimal solution, the current solution and the reverse solution are searched simultaneously, and the optimal solution is selected by comparing the fitness values of the current solution and its reverse solution. The initial population can increase the diversity of the population^20^ by adding a reverse population through the reverse learning strategy, and the reverse population solving formula is as follows:

12 $$\hat{X} = lb + ub - X$$

where $\hat{X}$ is the reverse solution.$X$ is the current solution.

Differential evolution algorithm^21^ comes from the genetic algorithm proposed earlier, and also has the evolution process of crossover, mutation and selection. Differential evolution of pelican population after reverse learning is carried out as follows:

First, each pelican individual of the current population and the reverse population was subjected to a mutation operation by Eq. (13) to obtain mutant individuals.

13 $$u_{i} = x_{i} + K\left( {x_{r1} - x_{r2} } \right)$$

where $x_{i}$ is the current pelican individual; $u_{i}$ is the mutant individual corresponding to the current pelican individual; K is the scaling factor; $x_{r1}$, $x_{r2}$ are two pelican individuals randomly selected.

Then, a new pelican individual is generated by the crossover operation of Eq. (14).

14 $$v_{i,j} = \left\{ \begin{array}{ll} u_{i,j} , & \quad rand\left( {0,1} \right) \le CR \hfill \\ x_{i,j} , & \quad otherwise \hfill \\ \end{array} \right.$$

where CR is the crossover probability.

Finally, more suitable individual pelicans were selected by comparing the magnitude of the fitness values, as shown in Eq. (15).

15 $$x_{i} = \left\{ \begin{array}{ll} v_{i} , & \quad if \, f\left( {v_{i} } \right){ <}f\left( {x_{i} } \right) \hfill \\ x_{i} , & \quad otherwise \hfill \\ \end{array} \right.$$

#### Optimal individual adaptive t-distribution variation

The convergence of the algorithm to the local extremum depends on the optimal position of the individual^22^. Therefore, in this paper, the adaptive t-distribution variation strategy in the literature^23^ is applied to the variation of the optimal pelican individual, and the current number of iterations is used as the degree of freedom of the t-distribution. At the beginning of the iteration, the t-distribution variation is close to the Coasean distribution variation, which is conducive to enhancing the search ability of the pelican individual at the global level and increasing the diversity of the population; at the end of the iteration, the t-distribution variation is close to the Gaussian distribution variation, which can enhance the search ability of pelican individuals near the optimal point and accelerate the convergence speed of the algorithm. The optimal individual adaptive t-distribution variance is formulated as follows:

16 $$X_{best}^{t} = X_{best} + X_{best} *t\left( {iter} \right)$$

where $X_{best}^{t}$ are mutated pelican individuals; $X_{best}$ are currently the best pelican individuals; iter is the number of current iterations; t represents t-distribution.

### Adaptive ε constraint method

The ε-constraint method^24^ is a method proposed by Takahama for solving constrained optimization problems, which retains infeasible individuals with low constraint violation and low objective function values by relaxing the constraints, and gives these excellent solutions a chance to enter the next generation population, which in turn explores to more regions and finds better objective function values. To overcome the problem that the traditional ε-constraint method tends to fall into local optimal solutions, an adaptive ε-constraint method is proposed in the literature^14^ with the following improvements:

1. Improving the individual comparison criterion to make full use of good infeasible individuals to explore to more solution space, thus increasing the population diversity. The specific criterion is shown in Eq. (17):
  17 $$X_{1} {\text{ is better than }}X_{2} \Leftrightarrow \left\{ \begin{aligned} & f\left( {X_{1} } \right) < f\left( {X_{2} } \right),G\left( {X_{1} } \right) = 0,G\left( {X_{2} } \right) = 0 \hfill \\ & f\left( {X_{1} } \right) < f\left( {X_{2} } \right),0 < G\left( {X_{1} } \right) \le \varepsilon ,0 < G\left( {X_{2} } \right) \le \varepsilon \hfill \\ & 0 < G\left( {X_{1} } \right) \le \varepsilon ,G\left( {X_{2} } \right) > \varepsilon \hfill \\ & G\left( {X_{1} } \right) < G\left( {X_{2} } \right),G\left( {X_{1} } \right) > \varepsilon ,G\left( {X_{2} } \right) > \varepsilon ,rand \le Ps \hfill \\ & f\left( {X_{1} } \right) < f\left( {X_{2} } \right),G\left( {X_{1} } \right) > \varepsilon ,G\left( {X_{2} } \right) > \varepsilon ,rand \le Ps \hfill \\ & f\left( {X_{1} } \right) < f\left( {X_{2} } \right),G\left( {X_{1} } \right) = 0,0 < G\left( {X_{2} } \right) \le \varepsilon \hfill \\ & G\left( {X_{1} } \right) = 0,G\left( {X_{2} } \right) > \varepsilon \hfill \\ &f\left( {X_{1} } \right) < f\left( {X_{2} } \right),0 < G\left( {X_{1} } \right) \le \varepsilon ,G\left( {X_{2} } \right) = 0 \hfill \\ \end{aligned} \right.$$
  where f(x) is the function fitness value;G(x) is the constraint violation value; Ps is a random number on the interval [0.9,1].
2. Adaptive ε adjustment strategy, the ε value of the traditional ε constraint method depends only on the number of iterations, the adaptive ε value fully considers the relationship between the objective function value and the size of constraint violation and the proportion of feasible individuals in the population, and makes adaptive adjustments at each generation. ε equation is as follows:
  18 $$\varepsilon \left( t \right) = \left\{ \begin{array}{ll} \varepsilon \left( 0 \right) \times e^{{ - \alpha \times \left( {t/Te} \right)}} , & \quad t \le Te \hfill \\ 0, & \quad t > Te \hfill \\ \end{array} \right.$$
  19 $$\varepsilon \left( 0 \right) = 0.6 \times \sum\limits_{i = 1}^{N} {G\left( {X_{i} } \right)/N}$$
  20 $$\alpha = \alpha_{\min } + \lambda \times \left( {\alpha_{\max } - \alpha_{\min } } \right)$$
  where Te is the number of truncated evolutionary iterations;$\lambda$ is the proportion of viable individuals in the population. The value of Te should be appropriate, too large will make a large number of infeasible individuals appear in the late iteration and affect the population convergence to a feasible solution; too small will eliminate a large number of infeasible individuals in the early iteration and easily fall into a local optimum solution.

### Computational flow of the ε-IPOA algorithm

**Figure 2:**
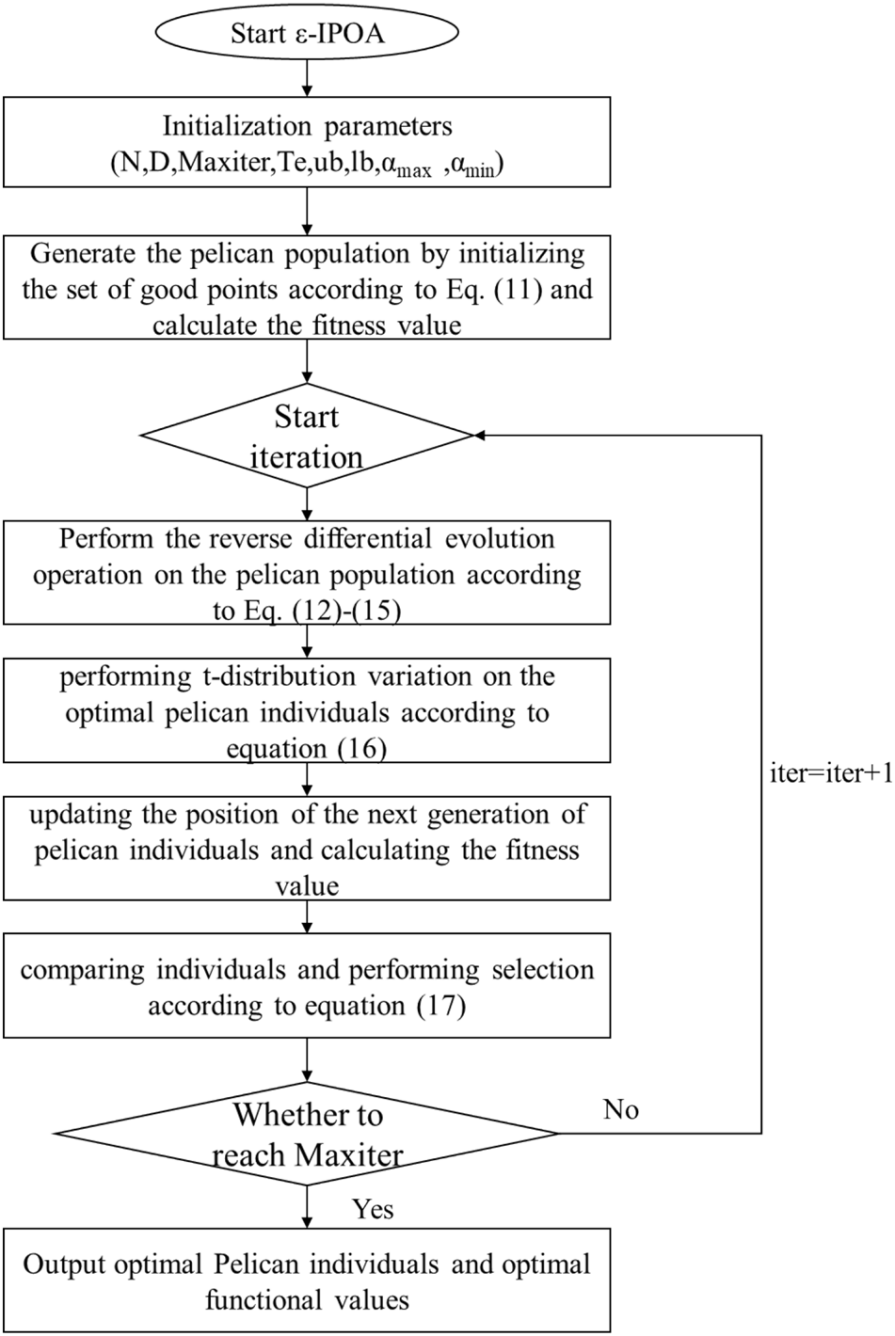
ε-IPOA algorithm flow chart.

The steps of the ε-IPOA algorithm are as follows, and the flowchart is shown in Fig. 2.

Step 1: Initialize the parameters, including the number of populations N, the maximum number of iterations T, the number of truncated evolutionary iterations Te, $\alpha_{\min }$, $\alpha_{\max }$, the boundary conditions and the dimensionality of the decision variables.
Step 2: Generate the pelican population by initializing the set of good points according to Eq. (11) and calculate the fitness value.
Step 3: Perform the reverse differential evolution operation on the pelican population according to Eqs. (12)–(15).
Step 4: Performing t-distribution variation on the optimal pelican individuals according to Eq. (16).
Step 5: Updating the position of the next generation of pelican individuals and calculating the fitness value.
Step 6: Comparing individuals and performing selection according to Eq. (17).
Step 7: Determine whether the condition to terminate the iteration is met, if so, go to step 8, otherwise go to step 3.
Step 8: Output the optimal pelican individual and the optimal fitness value.

### Simulation test of ε-IPOA algorithm

To verify the effectiveness and feasibility of the POA algorithm, this article selects 24 test functions from the internationally recognized cec2006 test function set for simulation experiments, and compares them with DE and POA algorithms. Among them, each test function runs independently 50 times, with a population of 200, a maximum iteration count of 10,000, a maximum function evaluation count of 500,000, a truncated evolution iteration count of 1000, and a tolerance value of 0.0001 for equation constraint violations. The experimental results are shown in Table 3.

The experimental results are shown in the table above, and the bolded font indicates the optimal effect. From Table 1, it can be seen that:

1. Adaptive ε-constraint method can effectively assist the IPOA algorithm in handling constrained optimization problems.
2. The three algorithms run independently on each function for 50 times. When the evaluation times are consistent, the average value of the results obtained by ε-IPOA algorithm is lower than that of the other two algorithms. This shows that when the Time complexity is consistent, the performance of ε-IPOA algorithm is better than that of ε-POA algorithm and ε-DE algorithm, and it has stronger global search ability. Except for the g20 and g22 functions, ε-IPOA algorithm has found the optimal solution that satisfies the constraint conditions.
3. The three algorithms were independently run 50 times on each function, and the standard deviation of the results obtained by ε-IPOA algorithm was smaller than that of the other two algorithms, demonstrating the good robustness of ε-IPOA algorithm.
4. ε-IPOA algorithm only achieves a standard deviation of 0 when solving functions g01 and g12, indicating that this algorithm still has potential for development and needs to further improve its exploration and development capabilities.

**Table 1:** Comparison results of constraint algorithms.

Function	Algorithm	Theoretical optimal value	Worst	Best	Mean	SD
g01	ε-IPOA	− 1.5000E+01	**− 1.5000E+01**	**− 1.5000E+01**	**− 1.5000E+01**	**0.0000E+00**
	ε-POA		− 1.4503E+01	− 8.1475E+00	− 1.1749E+01	1.6826E+00
	ε-DE		− 1.5000E+01	− 1.2453E+01	− 1.4715E+01	7.4398E−01
g02	ε-IPOA	− 8.0360E−01	**− 8.0360E−01**	**− 7.6875E−01**	**− 7.9333E−01**	**9.4435E−03**
	ε-POA		− 7.7833E−01	− 5.7747E−01	− 7.0594E−01	5.1016E−02
	ε-DE		− 6.5153E−01	− 2.7445E−01	− 4.5905E−01	1.2324E−01
g03	ε-IPOA	− 1.0005E+00	**− 1.0005E+00**	**− 1.0005E+00**	**− 1.0005E+00**	**2.3125E−06**
	ε-POA		− 8.5033E−01	− 5.1641E−02	− 3.4810E−01	2.1688E−01
	ε-DE		− 4.7602E−01	− 7.8962E−03	− 1.7354E−01	1.2715E−01
g04	ε-IPOA	− 3.0666E+04	**− 3.0666E+04**	**− 3.0666E+04**	**− 3.0666E+04**	**1.1101E−11**
	ε-POA		− 3.0666E+04	− 3.0661E+04	− 3.0665E+04	1.1365E+00
	ε-DE		**− 3.0666E+04**	**− 3.0666E+04**	**− 3.0666E+04**	**1.1101E−11**
g05	ε-IPOA	5.1265E+03	**5.1265E+03**	**5.1265E+03**	**5.1265E+03**	**9.7019E−13**
	ε-POA		5.1265E+03	5.1266E+03	5.1265E+03	2.8167E−02
	ε-DE		5.1272E+03	6.0487E+03	5.4180E+03	3.5816E+02
g06	ε-IPOA	− 6.9618E+03	**− 6.9618E+03**	**− 6.9618E+03**	**− 6.9618E+03**	**1.8501E−12**
	ε-POA		**− 6.9618E+03**	**− 6.9618E+03**	**− 6.9618E+03**	**1.8501E−12**
	ε-DE		**− 6.9618E+03**	**− 6.9618E+03**	**− 6.9618E+03**	**1.8501E−12**
g07	ε-IPOA	2.4306E+01	**2.4306E+01**	**2.4306E+01**	**2.4306E+01**	**1.3039E−06**
	ε-POA		2.4732E+01	3.7891E+01	3.0215E+01	3.9248E+00
	ε-DE		2.4400E+01	2.4485E+01	2.4434E+01	2.4382E−02
g08	ε-IPOA	− 9.5800E−02	**− 9.5800E−02**	**− 9.5800E−02**	**− 9.5800E−02**	**1.9962E−17**
	ε-POA		− 9.5825E−02	− 9.5825E−02	− 9.5825E−02	2.2760E−17
	ε-DE		− 9.5825E−02	− 2.7263E−02	− 9.1254E−02	1.7395E−02
g09	ε-IPOA	6.8063E+02	**6.8063E+02**	**6.8063E+02**	**6.8063E+02**	**4.6874E−13**
	ε-POA		6.8065E+02	6.8422E+02	6.8103E+02	7.3283E−01
	ε-DE		6.8063E+02	6.8064E+02	6.8064E+02	1.9027E−03
g10	ε-IPOA	7.0492E+03	**7.0492E+03**	**7.0492E+03**	**7.0492E+03**	**2.6164E−12**
	ε-POA		7.0953E+03	7.6499E+03	7.3103E+03	1.5389E+02
	ε-DE		7.1601E+03	7.4690E+03	7.2863E+03	5.7660E+01
g11	ε-IPOA	7.4990E−01	**7.4990E−01**	**7.4990E−01**	**7.4990E−01**	**1.1292E−16**
	ε-POA		7.4990E−01	7.4993E−01	7.4990E−01	5.5128E−06
	ε-DE		7.4990E−01	7.5635E−01	7.5014E−01	1.1789E−03
g12	ε-IPOA	− 1.0000E+00	**− 1.0000E+00**	**− 1.0000E+00**	**− 1.0000E+00**	**0.0000E+00**
	ε-POA		**− 1.0000E+00**	**− 1.0000E+00**	**− 1.0000E+00**	**0.0000E+00**
	ε-DE		**− 1.0000E+00**	**− 1.0000E+00**	**− 1.0000E+00**	**0.0000E+00**
g13	ε-IPOA	5.3900E−02	**5.3900E−02**	**4.3880E−01**	**2.9769E−01**	**1.8863E−01**
	ε-POA		5.4117E−02	8.6592E−01	3.8025E−01	2.0604E−01
	ε-DE		4.5505E−01	3.6967E+00	1.1278E+00	6.1815E−01
g14	ε-IPOA	− 4.7765E+01	**− 4.7765E+01**	**− 4.7765E+01**	**− 4.7765E+01**	**3.1307E−04**
	ε-POA		− 4.7744E+01	− 4.6145E+01	− 4.7294E+01	3.8388E−01
	ε-DE		− 4.6101E+01	− 3.5636E+01	− 4.1453E+01	2.5064E+00
g15	ε-IPOA	9.6172E+02	**9.6172E+02**	**9.6172E+02**	**9.6172E+02**	**1.4729E−11**
	ε-POA		9.6172E+02	9.6172E+02	9.6172E+02	1.5223E−05
	ε-DE		9.6172E+02	9.7044E+02	9.6388E+02	2.6095E+00
g16	ε-IPOA	− 1.9052E+00	**− 1.9052E+00**	**− 1.9052E+00**	**− 1.9052E+00**	**6.7752E−16**
	ε-POA		− 1.9052E+00	− 1.8994E+00	− 1.9049E+00	1.0415E−03
	ε-DE		− 1.9052E+00	− 1.9052E+00	− 1.9052E+00	8.5952E−14
g17	ε-IPOA	8.8535E+03	**8.8535E+03**	**8.9276E+03**	**8.8807E+03**	**3.6298E+01**
	ε-POA		8.8567E+03	8.9644E+03	8.8898E+03	4.4754E+01
	ε-DE		8.8735E+03	9.2980E+03	9.0170E+03	1.1064E+02
g18	ε-IPOA	− 8.6600E−01	**− 8.6600E−01**	**− 8.6600E−01**	**− 8.6600E−01**	**1.2602E−08**
	ε-POA		− 8.6423E−01	− 5.0218E−01	− 7.9447E−01	1.1080E−01
	ε-DE		− 8.6594E−01	− 8.6255E−01	− 8.6507E−01	6.9154E−04
g19	ε-IPOA	3.2656E+01	**3.2656E+01**	**3.2849E+01**	**3.2679E+01**	**4.5488E−02**
	ε-POA		3.4217E+01	6.3203E+01	4.0705E+01	7.0193E+00
	ε-DE		3.3697E+01	3.6170E+01	3.4753E+01	5.1021E−01
g20	ε-IPOA	2.0490E−01	–	–	–	–
	ε-POA		–	–	–	–
	ε-DE		–	–	–	–
g21	ε-IPOA	1.9372E+02	**1.9372E+02**	**1.9372E+02**	**1.9372E+02**	**8.6506E−12**
	ε-POA		8.3662E+01	3.1422E+02	2.0519E+02	5.3327E+01
	ε-DE		1.9375E+02	1.0000E+03	5.2062E+02	3.9881E+02
g22	ε-IPOA	2.3643E+02	–	–	–	–
	ε-POA		–	–	–	–
	ε-DE		–	–	–	–
g23	ε-IPOA	− 4.0006E+02	**− 4.0006E+02**	**− 4.0006E+02**	**− 4.0006E+02**	**2.1880E−04**
	ε-POA		− 8.1813E+00	− 6.1885E−02	− 4.0419E−01	1.4737E+00
	ε-DE		− 1.6037E+02	9.0000E+02	4.5899E+02	4.4110E+02
g24	ε-IPOA	− 5.5080E+00	**− 5.5080E+00**	**− 5.5080E+00**	**− 5.5080E+00**	**1.8067E−15**
	ε-POA		**− 5.5080E+00**	**− 5.5080E+00**	**− 5.5080E+00**	**1.8067E−15**
	ε-DE		**− 5.5080E+00**	**− 5.5080E+00**	**− 5.5080E+00**	**1.8067E−15**

## Case analysis

### Study area

**Figure 3:**
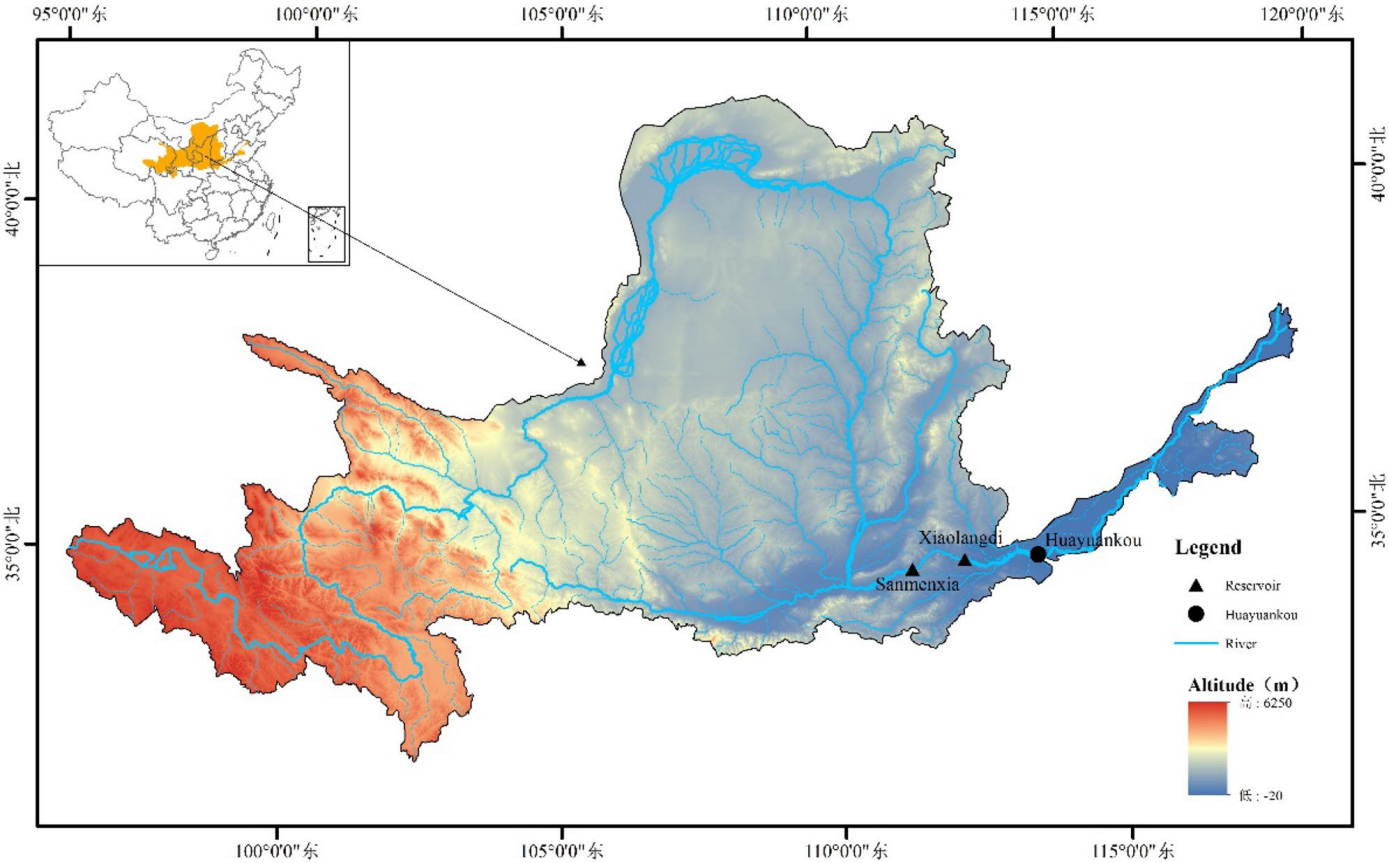
Geographical location map of the research object.

The Yellow River is the second largest river in China, with a total field of 5464 km and a basin area of 795,000 km^2^, originating from the Bayankara Mountains on the Qinghai-Tibet Plateau, flowing from west to east that through nine provinces and regions, including Qinghai, Sichuan, Gansu, Ningxia, Inner Mongolia, Shanxi, Shaanxi, Henan and Shandong, and injecting into the Bohai Sea in Kenli County, Shandong Province. In this paper, the area from Sanmenxia to Huankou in the middle and lower reaches of the Yellow River is selected as the study area, and flood control and scheduling research is carried out for Sanmenxia and Xiaolangdi tandem reservoirs. The geographical location is shown in Fig. 3. The tandem reservoir flood control and scheduling model takes the storage capacity of each reservoir at each time period as the decision variable, contains constraint constraints such as water balance, safe river discharge and gate discharge flow, and is solved by the ε-IPOA algorithm.

Sanmenxia Reservoir and Xiaolangdi Reservoir are both backbone reservoirs on the main stream of the middle and lower reaches of the Yellow River, mainly for flood control, taking into account the role of irrigation and power generation. Sanmenxia Reservoir controls a basin area of 688,000 km^2^, accounting for 91.5% of the total basin area of the Yellow River, and controls the flooding in the area above Sanmenxia. Xiaolangdi Reservoir controls a watershed area of 694,000 km^2^, accounting for 92.3% of the total area of the Yellow River basin, and is the only large comprehensive water conservancy project with large reservoir capacity in the middle and lower reaches of the Yellow River, except for Sanmenxia. The characteristic parameters of the two reservoirs are shown in Table 2.

**Table 2:** Reservoir characteristics parameters.

Parameters	Sanmenxia	Xiaolangdi
Flood limit water level (m)	307	230
Flood control high water level (m)	320	275
Flood control storage capacity (10^8^ m^3^)	59.79	40.5

### Flood process analysis

The floods in the area from Sanmenxia to Huayuankou mainly come from the upstream water of Sanmenxia reservoir, the floods between Sanmenxia and Xiaolangdi reservoir area and the floods between Xiaolangdi reservoir and Huayuankou control point. These three kinds of floods rise fiercely with high peaks and large amounts, causing a great threat to the downstream. In this paper, we select the 1000-year flood of 1958, which is the largest flood in the Yellow River since the availability of measured hydrological data, mainly caused by persistent heavy rainfall. The flood process is shown in Table 3.

**Table 3:** Flooding process.

Period of time	Inflow volume of Sanmenxia Reservoir (m^3^/s)	Interval flow of Sanmenxia-Xiaolangdi Reservoir (m^3^/s)	Interval flow of Xiaolangdi Reservoir-Huayuankou (m^3^/s)
1	9581	0	885
2	9581	0	885
3	7586	0	885
4	6636	0	885
5	6445	0	885
6	7348	0	885
7	9217	0	885
8	10,072	0	2302
9	9639	0	2442
10	9592	121	1289
11	8589	14,972	481
12	7516	10,441	183
13	7280	3994	0
14	7539	1565	0
15	7669	18,686	724
16	7787	20,554	0
17	8082	9422	2079
18	7834	5715	0
19	7716	1408	0
20	7716	1401	0
21	7669	1378	0
22	11,344	1355	0
23	12,638	1331	0
24	11,558	696	0
25	10,378	686	280
26	9198	683	2803
27	8316	2398	3131
28	7321	3305	2721
29	6554	1764	2833
30	5871	1764	1255
31	5573	1901	658
32	5402	1429	1019
33	5359	895	820
34	5288	758	758
35	5260	652	1205
36	5203	385	1541
37	4905	509	1416
38	4933	701	733
39	5527	394	745
40	5559	257	733
41	5606	536	0
42	5654	421	0
43	5701	604	0
44	5986	187	0
45	5939	0	0
46	5764	140	0
47	5669	536	0
48	5479	464	0
49	5226	323	0
50	4909	1047	0
51	4783	1302	0
52	5400	1370	0
53	5685	1438	0
54	5543	834	0
55	5147	370	0
56	4767	464	225
57	4561	1021	417
58	4688	1557	0
59	4957	1625	0
60	5004	1323	0
61	5289	1021	128
62	5986	489	302
63	8045	0	400
64	8916	0	494
65	9771	1625	332
66	10,769	1395	0
67	9375	417	0
68	7966	2323	0
69	7776	2595	0
70	7649	277	0
71	7221	47	528
72	7016	94	221
73	7237	0	123
74	7016	0	102
75	5780	1489	68
76	5084	2876	723
77	4513	3250	1089
78	4197	2689	123
79	3912	1808	0

**Figure 4:**
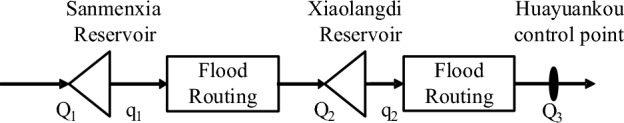
Flood evolution of Sanmenxia and Xiaolangdi reservoirs.

Figure 4 shows the flood evolution process from Sanmenxia reservoir to Huayuankou interval. It is assumed that the inlet flow of Sanmenxia reservoir is Q_1_, the interval flood from Sanmenxia to Xiaolangdi reservoir is Q_2_, and the interval flood from Xiaolangdi reservoir to the control point of Huayuankou is Q_3_. The flood process of Huayuankou section is composed of floods in each area through the action of river evolution and reservoir regulation.

The process of flood water moving from upstream to downstream in a river is called flood evolution. The study of flood evolution allows staff at downstream sites to forecast the flood process at downstream sites based on the flow process measured at upstream sites, providing a basis for downstream flood forecasting and flood control. The commonly used calculation methods are hydrological method and hydrodynamic method, the hydrological method is simple to calculate and requires less information, the hydrodynamics is limited by the measured topographic information^25^, this paper selects the hydrological method based on the tank storage equation and water balance principle—Muskingum method^26^ calculation, the parameter values used are shown in Table 4, Where K is the tank storage coefficient, x is the flow specific gravity coefficient, and △t is the time interval (hour).

**Table 4:** Muskingum parameters.

River section	Flood propagation time	Number of segments	K	△t	X
Sanmenxia–Xiaolangdi	8	2	3.875	4	0.2
Xiaolangdi–Huayuankou	12	3	4.567	4	0.3

## Results and discussion

This paper aims to minimize the peak flow of Huayuankou section, and adopts ε-IPOA algorithm solvethe model. In the solution, the population size and the maximum number of iterations are 200 and 30w, respectively, and the number of truncation iterations Te is 1000. based on the measured hydrological data, the flood calendar time and reservoir operation period in this paper are 13d, and the calculation period is 4 h.

### Analysis of operation results

**Figure 5:**
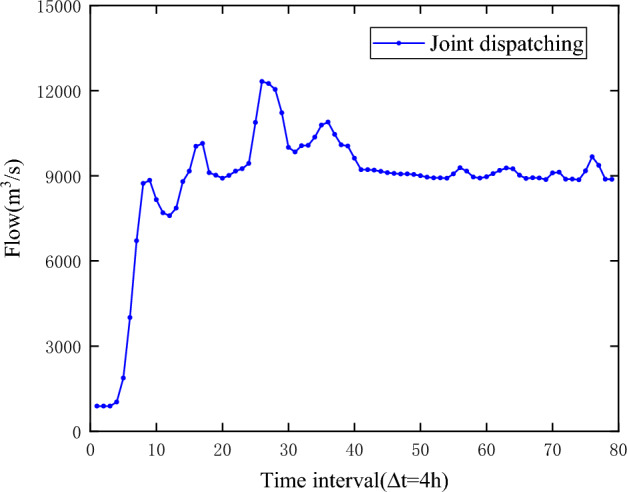
Huayuankou flood process.

**Figure 6:**
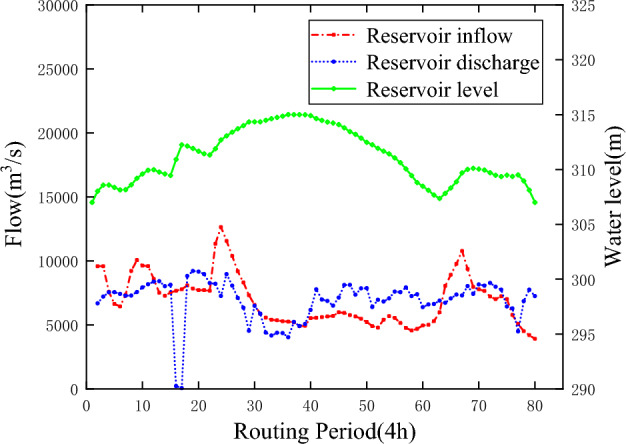
Sanmenxia Reservoir operation process.

**Figure 7:**
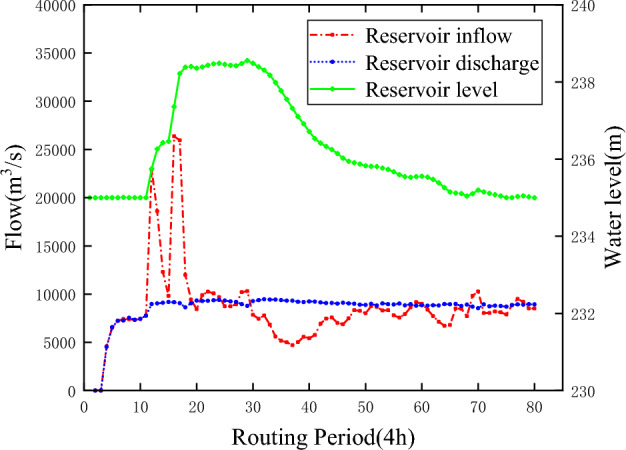
Xiaolangdi Reservoir operation process.

According to the maximum peak-clipping objective function of the garden mouth section established in “Objective function”, the ε-IPOA algorithm proposed in this paper is used to solve the flood control operation model of Sanmenxia and Xiaolangdi cascade reservoirs, and the flood process of the Huayuankou section is shown in Fig. 5 and Table 5, and the operation results of Sanmenxia and Xiaolangdi reservoirs are shown in Table 6, Figs. 6 and 7.

**Table 5:** Peak flow at Huayuankou.

Control station	Control peak flow (m^3^/s)	Peak flow (m^3^/s)	Peak clipping rate
Huayuankou	22,000	12,319	44%

**Table 6:** Calculation results of each reservoir.

Reservoir	Initial water level (m)	End water level (m)	Inflow peak flow (m^3^/s)	Discharge peak flow (m^3^/s)	Peak clipping rate (%)
Sanmenxia	307	307	12,638	9214	27.1
Xiaolangdi	235	235	26,376	9462	64.1

It can be seen from Tables 6 and 7 that after the joint dispatching, the peak flow of Huayuankou section is 12,319 m^3^/s, which does not exceed the controlled discharge of 22,000 m^3^/s of Huayuankou, and the peak clipping rate has reached 44%. Through the joint operation of the two reservoirs, the peak clipping rate have reached 27.1% and 64.1% respectively, which is conducive to the safety of the reservoir itself and reduces the flood control pressure in the downstream. Figures 6 and 7 are the operation process charts of Sanmenxia Reservoir and Xiaolangdi Reservoir respectively. It can be seen from the figure that the water level processes of the two reservoirs are between the flood limit water level and the flood control high water level, indicating that the solution results meet the constraints and reach the feasible solution. The inflow flood of Xiaolangdi Reservoir has reached a "double peak", with the maximum peak flow exceeding 25,000 m^3^/s, which greatly exceeds the safe discharge of the river channel and increases the risk of flood disaster. After regulation, the discharge flow of Xiaolangdi Reservoir is stable within 10,000 m^3^/s, and the flood process is generally stable, ensuring the safety of flood discharge of the downstream river channel.

**Table 7:** Comparison of operation results.

Operation mode	Control peak flow (m^3^/s)	Peak flow (m^3^/s)	Peak clipping rate (%)
Joint operation	22,000	12,319	44
Single reservoir operation	22,000	12,978	41

### Comparison of operation results

#### Algorithm comparison

**Figure 8:**
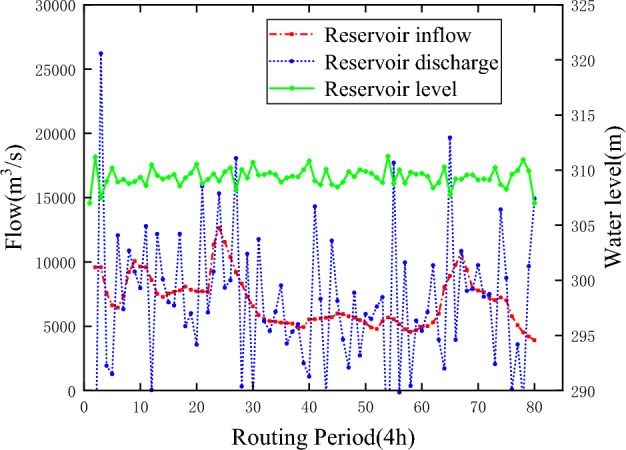
ε-POA solves the scheduling process of Sanmenxia reservoir.

**Figure 9:**
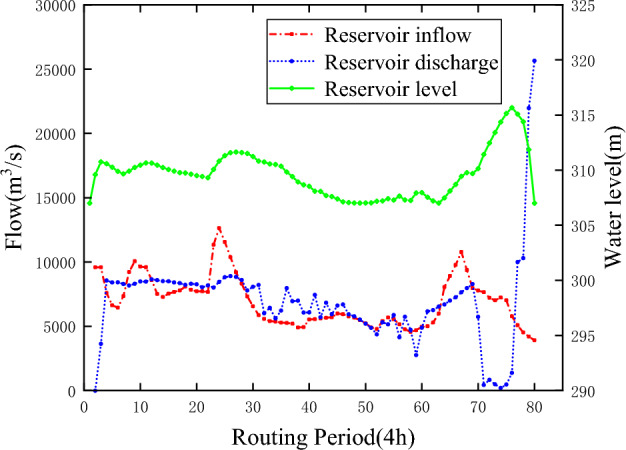
ε-DE solves the scheduling process of Sanmenxia reservoir.

To verify the feasibility and applicability of the ε-IPOA algorithm, the ε-POA and ε-DE algorithms are chosen to solve the above model in this paper. To ensure the fairness of the algorithms, the parameters and initial conditions of the ε-POA and ε-DE algorithms are kept the same as those of the ε-IPOA algorithm. Unfortunately, neither algorithm found a feasible solution for the model, further illustrating the superiority of the ε-IPOA algorithm in solving the reservoir scheduling problem. Figures 8 and 9 show the solution results of the ε-POA and ε-DE algorithms for the Sanmenxia reservoir, respectively.

#### Comparison with single reservoir scheduling results

In order to illustrate more intuitively the effect of joint operation than single reservoir operation, this paper also calculates the flooding process of the Huayuankou section under single reservoir operation, and the results are shown in Tables 7 and 8.

It can be seen from the above table that under the two modes, the peak flow of Huayuankou section does not exceed the control flow, but the peak clipping effect of Huayuankou section under the joint dispatching mode is better than that of single reservoir dispatching. By comparing Tables 6 and 8, under the single reservoir operation mode, the water levels of Sanmenxia and Xiaolangdi reservoirs have not recovered to the initial water level at the end of the operation period, which is not conducive to coping with the arrival of the next flood in the flood season. In addition, under the single reservoir operation mode, the peak clipping rate of Sanmenxia and Xiaolangdi reservoirs is lower than that of joint operation. Therefore, the peak clipping effect of the joint operation mode is the best, which can play a more important role in flood control of the reservoir.

**Table 8:** Single reservoir operation results.

Reservoir	Initial water level (m)	End water level (m)	Inflow peak flow (m^3^/s)	Discharge peak flow (m^3^/s)	Peak clipping rate (%)
Sanmenxia	307	314	12,638	9878	21.8
Xiaolangdi	235	241	26,376	12,000	57.3

## Conclusion

In this paper, the adaptive ε-constrained method is coupled with the Pelican algorithm, and then the ε-IPOA algorithm is proposed by initializing the population with good point set, reverse differential evolution and optimal individual adaptive strategy to improve the Pelican algorithm. The case of Sanmenxia and Xiaolangdi reservoirs is also selected, and the joint flood control operation model of the cascade reservoirs is established based on the maximum peak clipping criterion, and this algorithm is applied to the solution of the model. The conclusions are as follows.

1. Select 24 test functions from the cec2006 test set to validate the ε-IPOA algorithm and compare them with ε-DE and ε-POA algorithms. The experimental results show that the ε-IPOA algorithm is superior to the other two algorithms, with stable solving performance and high accuracy, and can effectively handle constrained optimization problems. The Pelican optimization algorithm can improve its global search ability by initializing the population with a good point set, reverse difference mixing, and perturbing the optimal individual t-distribution.
2. In this paper, the ε-IPOA algorithm is used to solve the cascade reservoirs flood control operation problem, and the results obtained satisfy all the constraints, and the peak clipping rates of Sanmenxia reservoir, Xiaolangdi reservoir and Huayuankou control point are 27.1%, 64.1% and 44%, respectively, which effectively ensure the flood safety of the Huayuankou section. And the ε-POA algorithm and ε-DE algorithm with which for did not find a feasible solution. In addition, the results of the optimal operation of single reservoir are compared, and the joint operation is better than the single reservoir operation.
3. The results indicate that the ε-IPOA algorithm is feasible for solving the optimization operation problem of reservoir flood control, and can effectively address the strong constraints, multi-stage, and high-dimensional problems of reservoir operation models. This algorithm provides a new approach to solve the optimization scheduling problem of reservoir groups.
4. In the future, the performance of this algorithm will be further optimized through other strategies and applied to more complex series parallel hybrid reservoir groups. In addition, this algorithm is also applied to other engineering constrained optimization problems.

## Data Availability

The datasets generated and/or analyzed during the current study are not publicly available but are available from the corresponding author on reasonable request.
